# System Pharmacology-Based Strategy to Decode the Synergistic Mechanism of GanDouLing for Wilson's Disease

**DOI:** 10.1155/2021/1248920

**Published:** 2021-01-29

**Authors:** Juan Zhang, Hong Chen, Yuancheng Bao, Daojun Xie, Wenming Yang, Huaizhou Jiang, Ting Dong, Hui Han

**Affiliations:** ^1^Encephalopathy Center, The First Affiliated Hospital of Anhui University of Chinese Medicine, 117 Meishan Road, Shushan District, Hefei 230031, China; ^2^Graduate School, Anhui University of Chinese Medicine, 103 Meishan Road, Shushan District, Hefei 230038, China; ^3^Basic Department of Traditional Chinese Medicine, Anhui University of Chinese Medicine, 103 Meishan Road, Shushan District, Hefei 230038, China

## Abstract

**Results:**

Firstly, 324 active compounds have been identified in the GDL formula. Meanwhile, we identified 1496 human genes which are related to WD or liver cirrhosis. Functional and pathway enrichment analysis indicated that NOD-like receptor signaling pathway, bile secretion, calcium signaling pathway, steroid hormone biosynthesis, T cell receptor signaling pathway, apoptosis, MAPK signaling pathway, and so forth can be obviously regulated by GDL. Further, in a mouse model of WD, in vivo experiments showed that GDL treatment can not only reduce the pathological symptoms of the liver but also reduce the apoptosis of hepatocytes.

**Conclusions:**

In this study, systemic pharmacological methods were proposed and the mechanism of GDL combined therapy for WD was explored. This method can be used as a reference for the study of other mechanisms of traditional Chinese medicine.

## 1. Introduction

Wilson's disease (WD) is a hereditary disease which was first proposed by Kinnear Wilson in 1912 and caused by mutations in the ATP7B copper transporter gene that causes copper to accumulate in the liver and brain [1]. The phenotypic manifestations of WD are often highly variable, involving varying degrees of liver and/or neurological symptoms [2–4]. There is increasing evidence showing that the clinical manifestations of WD are affected by genetic and epigenetic factors [5–7], which may be responsible for the high phenotypic variability of WD. WD's liver damage ranges from mildly symptomatic to steatosis, cirrhosis, or acute liver failure. The involvement of the nervous system is characterized by dyskinesia, manifested as Huntington's disease-like tonic and tremor.

According to its pathogenesis, WD is treated by blocking intestinal copper uptake by chelating agents, such as triene amine, tetrathiomolybdate, or zinc salts. However, their high cost or side effects may hinder clinical use [8]. At present, penicillamine (d-penicillamine) is widely used, because of its low cost and remarkable curative effect [9, 10]. But, with many adverse reactions, such as fever, rash, gastrointestinal reaction, leukopenia, systemic lupus erythematosus, and nephritis, which limit its clinical application [11], a large number of studies have shown that *Penicillium* has therapeutic effects on liver diseases [12, 13], especially WD [9, 13]. A large number of clinical practice and theoretical studies have proved that traditional Chinese medicine is an effective drug for the treatment of WD. Traditional Chinese medicine can eliminate the abnormal deposition of copper ions in the liver, brain, and other tissues through not only kidney metabolic pathway but also bile secretion, so as to improve the curative effect and reduce side effects. More importantly, the combination of traditional Chinese and Western medicine can reduce side effects. GanDouLing is a TCM, and as an in-hospital preparation, it was prepared by the First Affiliated Hospital of Anhui University of Traditional Chinese Medicine [14, 15], which has proved that there can be effective improvement of liver and neuropathy in clinical, animal, and cell models [16–19]. Specifically, it can effectively play an important role in removing blood stasis from the liver and gallbladder, promoting blood circulation and removing copper. GanDouLing is mainly composed of *Gynochthodes officinalis* (F.C.How) Razafim. & B.Bremer (Bajitian, BJT), *Schisandra chinensis* (Turcz.) Baill (Beiwuweizi, BWWZ), *Rheum officinale* Baill (Dahuang, DH), *Curcuma zedoaria* (Christm.) Roscoe (Ezhu, EZ), *Coptis chinensis* Franch (Huanglian, HL), *Curcuma longa* L. (Jianghuang, JH), *Lysimachia christinae* Hance (Jinqiancao, JQC), and *Rehmannia glutinosa* (Gaertn.) DC. (Shudihuang, SDH) [20, 21].

Network pharmacology is an emerging discipline, which is based on the characteristics of biomolecules and a number of authoritative databases showing us a preliminary understanding of mechanisms of medicine and disease. The previous research study used the method of network pharmacology to provide a promising method for understanding the pharmacological mechanism of TCM. In the article, we developed a network pharmacology analysis to determine the anti-WD mechanism of GDL and the experiments in vitro were then performed given the results of network pharmacology analysis.

## 2. Methods

### 2.1. Chemical Components Database, ADME Screening, and Targets Identification

GanDouLing is a TCM, and as an in-hospital preparation, it was prepared by the First Affiliated Hospital of Anhui University of Traditional Chinese Medicine and the quality control of chemical fingerprint has been performed [14, 15], which is mainly composed of BJT, BWWZ, DH, EZ, HL, JH, JQC, and SDH [20, 21]. Following with a strategy of system pharmacology, the pharmacology mechanisms of WD by GDL were encoded. Firstly, all compounds of GDL containing eight herbs were gathered from the TCMSP database (http://lsp.nwu.edu.cn/index.php) [22] with the parameter of oral bioavailability (OB) [23], drug-likeness (DL) [24], and so forth. Next, the active components were filtered out by the ADME method. Targets of the active components in GDL were predicted by BATMAN-TCM [25]; next, the intersection genes of GDL targets and WD-related genes including liver cirrhosis collected from the Comparative Toxicogenomics Database (CTD) [26] and DisGeNET [24, 27] were identified. Then, GO [28] and KEGG [29] pathway analysis was performed by clusterProfiler [30]. Last, the PPI network was constructed by STRING [31] and visualized by Cytoscape [32], and the modules of PPI were identified by MCODE [33].

### 2.2. Establishment of Animal Models

Twenty female TX mice (20 ± 2 g) and ten female DL mice were at the ages of 8–10 weeks [34]. Original mice were obtained from the Jackson laboratory with the help of Beijing Vital River Laboratory Animal Technology, Ltd. This study was carried out in strict accordance with the “National Institutes of Health Guidelines for the Care and Use of Experimental Animals.” It was approved by the Institutional Animal Care and Use Committee of the Anhui Hospital of TCM.

The twenty female TX mice were divided into two groups, 10 mice in each group: the Wilson group and GanDouLing group (0.486 g/kg/day, GanDouLing was produced by the Anhui Hospital of TCM: the active ingredients of each herb were extracted with 65% ethanol and then combined with extracted filtrate; the filtrate combinations were baked into dry paste at the right temperature and starch was added and packed into GanDouLing troche) [16]. The ten female DL mice (20 ± 2 g) were treated as the control group. The mice in the GanDouLing groups were treated by intragastric administration with GanDouLing for 8 weeks. The Wilson group and control group were treated by intragastric administration with an equivalent volume of 0.9% saline every day. All the mice were housed in a controlled humidity (50–70%) and room temperature (18–22°C), fed in the isolation cages with independent air supply, and were given free access to food and water ad libitum in an alternating 12 h light/dark cycle over a period of 8 weeks.

### 2.3. Hematoxylin and Eosin Stain

The mice liver isolated tissues were fixed with 4% paraformaldehyde for 3 h and then dehydrated with ethanol and xylene. According to the previous literature, staining was carried out for liver samples [16]. The result of staining was that the cell nucleus was dyed blue by Hematoxylin and the cytoplasm was dyed red by Eosin.

### 2.4. Western Blot

According to the previous literature, HE staining was carried out for liver samples [16]. The details of antibodies are as follows: rabbit anti-B-cell lymphoma 2 (BCL-2) at 1 : 1000 (ab32124; Abcam, Cambridge, UK), rabbit anti-Bcl-2-associated X (BAX) at 1 : 1000 (ab32503; Abcam, Cambridge, UK), and mouse anti-GAPDH at 1 : 2000 (sc-32233; Santa Cruz, Santa Cruz, CA, USA), goat anti-mouse IgG H&L at 1 : 2000 (sc-2005; Santa Cruz, Santa Cruz, CA, USA), or goat anti-rabbit IgG H&L at 1 : 5000 (ab6721; Abcam, Cambridge, UK).

### 2.5. Assay of Caspase-3 Activity

Caspase-3 activity assay kit (BB-4106; BestBio, Shanghai, China) and Caspase-8 activity assay kit (BB-4107; BestBio, Shanghai, China) were used to detect the activity of caspase-3 and caspase-8 in three groups, according to the manufacturer's protocol.

### 2.6. Statistical Analysis

GraphPad Prism 7 was employed for comparing the expression of Bcl-2 and Bax and the activity of caspase-3 and caspase-8. And the method of Student's *t*-test was used for analyzing the comparison. When *P* < 0.05, it was considered statistically significant.

## 3. Results

Following a system pharmacology model, the pharmacology mechanisms of WD by GDL were encoded. Firstly, all compounds of GDL were gathered from a database. Next, the active components were filtered out by the ADME method. Then, the targets, pathway, and PPI network were identified from integrated predictive models. Finally, we employed the TX-j mouse model for proving that GDL could improve WD liver injury by regulating apoptosis.

### 3.1. Active Components Target for Anti-WD in GDL

**Figure 1:**
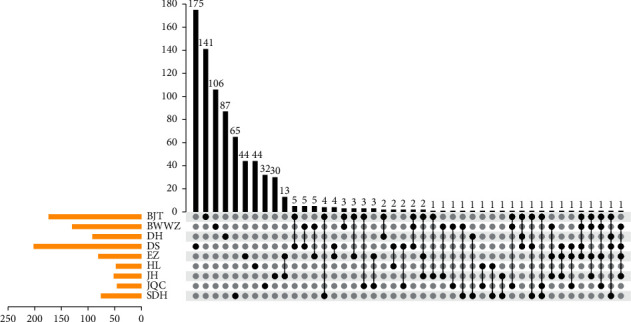
Upset chart showing the overlapping ingredients in each herb with GanDouLing.

**Figure 2:**
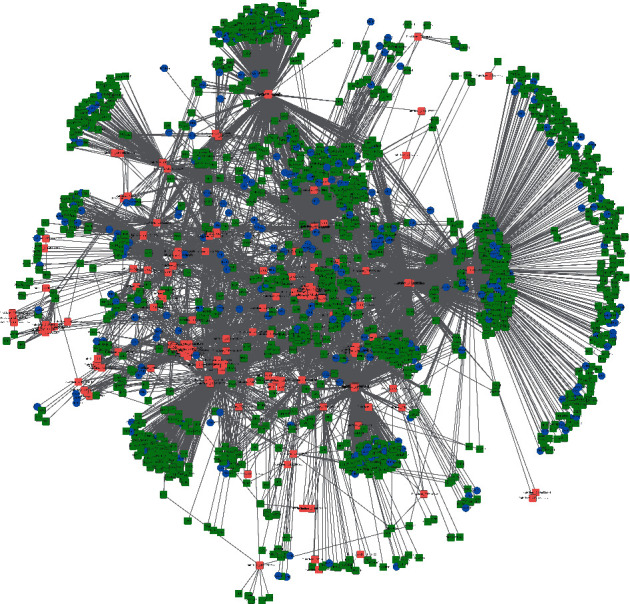
Ingredient-target network. Pink square represents active compounds in GanDouLing, green and blue nodes represent the target genes of active compounds, blue square represents common targets, and blue circles represent WD targets.

In total, 802 compounds were obtained for GDL, including 174 compounds for Bajitian, 130 compounds for Beiwuweizi, 92 compounds for Dahuang, 202 compounds for Danshen, 81 compounds for Ezhu, 48 compounds for Huanglian, 52 compounds for Jianghuang, 61 compounds for Jinqiancao, and 76 compounds for Shudihuang (Figure 1). Up to 65 compounds are contributed to more than one herb. By contrast, the other compounds belong to only one of the nine herbs. And then, two ADME properties of these components including OB and DL were focused to further state the components of GDL. Generally speaking, any TCM formulation consists of many components, but most of the components do not possess satisfactory properties of pharmacodynamic and pharmacokinetic. In the present study, OB and DL were adopted to filter for active components. After screening, 324 active components were screened out of the 802 components of GDL. And the targets for active components were predicted by BATMAN-TCM. In total, 1,496 genes were identified which are related to WD and liver cirrhosis, and 287 genes could be a target with 151 active components. And the components-target network was performed by Cytoscape (Figure 2).

### 3.2. GO and Pathway Analysis to Encode the Pharmacological Mechanisms of GDL

To explain the molecular biology of the therapy mechanism of GDL for WD, all target proteins were used to perform the enrichment of GO and KEGG pathways by clusterProfiler.

**Figure 3:**
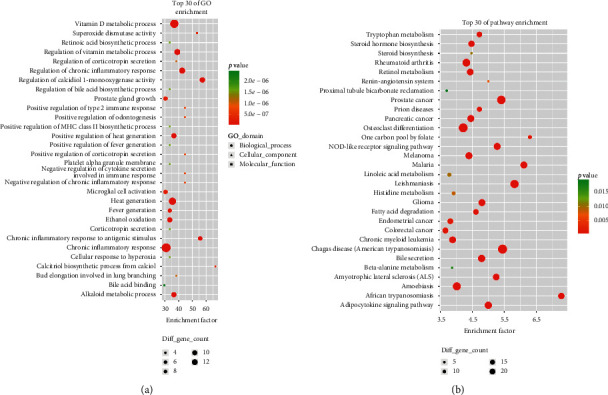
GO and pathway analysis of the targets of WD targets. (a) The top 30 significant GO terms. (b) The top 30 signaling pathways. The different colors from green to red represent the *P* value. The different sizes of the shapes represent the gene count number. The larger the proportion is, the larger the dot; the redder the dot color, the more significant the *P* value will be.

Enrichment analysis showed that a total of 2216 GO-terms with *P* < 0.05 were significantly enriched with 287 targets, 1915 of which were BP, 108 of which were CC, and 192 of which were MF. The top 10 enriched GO biological processes were response to organic cyclic compound (GO:0014070), response to hormone (GO:0009725), response to organic substance (GO:0010033), response to lipid (GO:0033993), response to organonitrogen compound (GO:0010243), regulation of cell proliferation (GO:0042127), response to steroid hormone (GO:0048545), response to drug (GO:0042493), cell proliferation (GO:0008283), and cellular response to organic substance (GO:0071310). In particular, the results show that targets are involved in the regulation of cell death (GO:0010941, 93 targets), apoptotic process (GO:0006915, 97 targets), inflammatory response (GO:0006954, 58 targets), and so forth. Extracellular space (GO:0005615), cell surface (GO:0009986), vesicle (GO:0031982), membrane-bounded vesicle (GO:0031988), extracellular region (GO:0005576), cytoplasmic vesicle (GO:0031410), cytoplasmic membrane-bounded vesicle (GO:0016023), secretory granule (GO:0030141), cytoplasmic membrane-bounded vesicle lumen (GO:0060205), and cytosol (GO:0005829) were the top 10 enriched cellular components. The enriched GO molecular functions were receptor binding (GO:0005102), oxidoreductase activity (GO:0016491), identical protein binding (GO:0042802), enzyme binding (GO:0019899), steroid binding (GO:0005496), transcription factor binding (GO:0008134), carboxylic acid binding (GO:0031406), organic acid binding (GO:0043177), antioxidant activity (GO:0016209), and iron ion binding (GO:0005506). The top 30 GO-terms with the highest enrichment factor are shown in Figure 3(a).

For 287 targets, a total of 176 pathway terms were enriched by KEGG enrichment analysis, and 43 KEGG pathway terms were significant with *P* < 0.05. The top 10 enriched pathways were Chagas disease (American trypanosomiasis) (hsa05142), prostate cancer (hsa05215), leishmaniasis (hsa05140), osteoclast differentiation (hsa04380), malaria (hsa05144), African trypanosomiasis (hsa05143), pathways in cancer (hsa05200), adipocytokine signaling pathway (hsa04920), rheumatoid arthritis (hsa05323), and NOD-like receptor signaling pathway (hsa04621). In particular, we found that targets are involved in the bile secretion (hsa04976, 12 targets), calcium signaling pathway (hsa04020, 18 targets), T cell receptor signaling pathway (hsa04660, 13 targets), fatty acid degradation (hsa00071, 7 targets), apoptosis (hsa04210, 10 targets), MAPK signaling pathway (hsa04010, 21 targets), cytokine-cytokine receptor interaction (hsa04060, 20 targets), PPAR signaling pathway (hsa03320, 8 targets), Toll-like receptor signaling pathway (hsa04620, 10 targets), ErbB signaling pathway (hsa04012, 9 targets), B cell receptor signaling pathway (hsa04662, 8 targets), Jak-STAT signaling pathway (hsa04630, 13 targets), and so forth. The top 30 pathway terms with the highest enrichment factors are shown in Figure 3(b).

### 3.3. PPI Network

**Figure 4:**
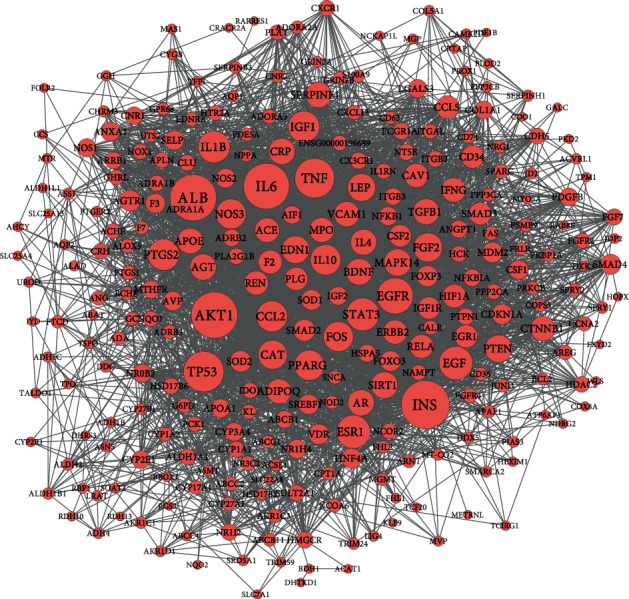
PPI network of WD targets. Red circles represent WD targets between ingredient targets from GanDouLing and WD significant targets.

The PPI network for the target genes was constructed based on the STRING database. As shown in Figure 4, the PPI network consists of 283 target genes and 4,332 edges, which means some target genes could interact with numerous genes. In fact, we found that dozens of target genes exhibited high degree characteristics, such as INS (insulin, degree = 160), ALB (albumin, degree = 152), AKT1 (AKT serine/threonine kinase 1, degree = 152), IL6 (interleukin 6, degree = 150), TP53 (tumor protein p53, degree = 128), TNF (tumor necrosis factor, degree = 125), and EGFR (epidermal growth factor receptor, degree = 111). High connectivity nodes with degree ≥70 are listed in Table 1.

**Figure 5:**
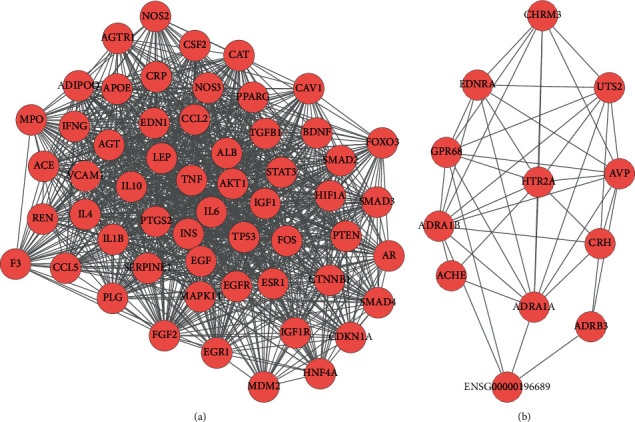
Two key modules network (a)-(b) of PPI network for GanDouLing target with WD.

The plug-in of MCODE was used to identify the modules with the PPI, and a total of 13 modules were gained. By sorting with MCODE score, two modules with MCODE score ≥3 and nodes ≥10 were screened out and named module 1 and module 2. And then, the subnetwork visualization was performed for module 1 and module 2 (Figure 5).

### 3.4. Effects of GDL on the Mouse Model of WD

**Figure 6:**
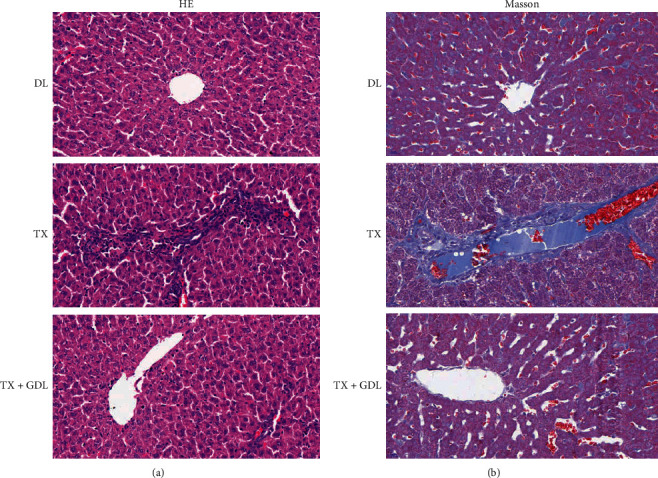
The liver injury induced by WD by HE (a) and Masson staining (b) in control (DL), WD (TX), and GanDouLing (TX + GDL) groups.

In order to observe the protection effects of GDL with WD, TX mice were treated by intragastric administration with GanDouLing in 0.486 g/kg every day for 8 weeks. The results indicated that WD induced liver injury in the mouse model. HE staining results shown in Figure 6 suggested that the control group presented healthy tissues, whereas the WD models presented a diffuse lesion in the liver. A large number of hepatic parenchymal cells were observed to be necrotic in TX compared with the control. And GDL could effectively improve these lesions.

Collagen was stained blue in liver sections with Masson staining. The TX mice showed obvious collagen accumulation compared with the control group (Figure 6(b)), and administration GanDouLing can reverse.

### 3.5. Effects of GDL on Protein Expressions and Caspase Activity Associated with Apoptosis

In the analysis of network pharmacology, we found that there are more than 102 targets for anti-WD with GDL enriched to cell death, and up to 10 targets for anti-WD with GDL were enriched to the apoptosis signaling pathway, including NFKB1, PPP3CA, APAF1, RELA, AKT1, PPP3CB, IL1B, NFKBIA, TNF, and BCL2.

**Figure 7:**
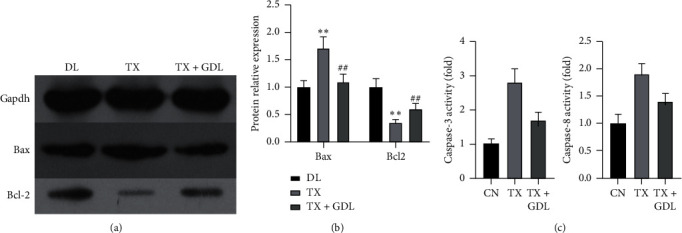
Effects of GanDouLing on protein expressions and caspase activity associated with apoptosis. (a)-(b) Western blotting result of Bcl-2 and Bax in the liver tissue. (c) The activity of Caspase-3 and Caspase-8 in the liver tissue.

In order to explore the possible mechanism for WD, effects of the GDL formula on apoptosis relative proteins including Bcl-2 and Bax were observed using the western blotting method. In comparison with the control group, Bax was obviously overexpressed and Bcl-2 was suppressed in the Wilson group, and GanDouLing could reverse the expression of Bax and Bcl-2 (Figures 7(a) and 7(b)). As shown in Figure 7(c), the TX group led to a 2.8-fold increase in caspase-3 and 1.9-fold increase in caspase-8 expression levels compared with controls. The above results indicated that GanDouLing can inhibit apoptosis of liver cells.

## 4. Discussion

At present, systemic pharmacology provides a powerful tool for encoding the compatibility and mechanism of traditional Chinese medicine [35–37]; there are many studies focusing on liver disease or injury based on the theory of systems pharmacology. On the basis of network pharmacology, the antioxidative effect of Zhizi Dahuang Decoction on alcoholic liver disease was expounded [38]. Wei et al. successfully revealed that 16 targets related to 11 compounds of SCG are closely related to the treatment of liver fibrosis, and TGF-*β* 1/Smad signal pathway is relatively important. They also demonstrated through animal experiments that SCG can significantly improve liver fibrosis by inhibiting TGF-*β* 1/Smad pathway [39]. In order to explain the pharmacological mechanism of Yinchenhao decoction (YCHD) which is a classical TCM formula that has been widely used in the treatment of liver fibrosis caused by chronic hepatitis B and jaundice for more than 1800 years, Cai et al. have employed the methods of network pharmacology and transcriptomic analysis to systematically describe the pharmacological mechanism [40]. At the same time, a large number of studies on the treatment of hepatocellular carcinoma with TCM have been carried out [41–43].

In this study, we performed the system pharmacology to construct a strategy for decoding the TCM pharmacologic molecular mechanism of GanDouLing which has proved that it can be an effective improvement of liver and neuropathy in clinical, animal, and cell models [16–19] With the integrated application of physical and chemical properties, network topological features, functional analysis, and path analysis provide a reference for the new method. However, its discovery mainly depends on theoretical analysis, so more experiments are needed to verify our findings and potential clinical significance. We find that some recent studies have introduced experiments such as transcriptome, proteomics, qPCR, and WB. [39, 40, 44]. In our study, with regular system pharmacology, we found that hundreds of GO-terms could be affected by GDL, such as cell proliferation, cell death [45], inflammatory response [46], response to oxidative stress [47], and response to lipid [48]. Meanwhile, dozens of pathways could be affected by GDL including adipocytokine signaling pathway, NOD-like receptor signaling pathway, bile secretion, retinol metabolism, calcium signaling pathway, steroid hormone biosynthesis, T cell receptor signaling pathway, apoptosis [45], MAPK signaling pathway, cytokine-cytokine receptor interaction, PPAR signaling pathway, Toll-like receptor signaling pathway, B cell receptor signaling pathway, Jak-STAT signaling pathway, and focal adhesion.

The TX-j mouse model was employed for proving that GDL could improve WD liver injury, by regulating apoptosis. Firstly, HE and Masson staining showed that GanDouLing could improve the necrosis and fibrosis of the TX-j liver tissue. Furthermore, targeting the apoptosis signaling pathway identified by network pharmacology, we found that GanDouLing significantly inhibited the expression of proapoptosis Bax protein and promoted the expression of antiapoptosis BCL2. In addition, the activity of caspase-3 and caspase-8 was tested and found to be significantly increased in TX-j mice, which was consistent with the previous research results: the activity of caspase-3 increased in WD patients or copper-induced hepatocytes [49, 50]. Meanwhile, GanDouLing can reduce its activity to a certain extent.

## 5. Conclusion

In conclusion, the mechanism of GDL in the treatment of WD involves a variety of active components, targets, and pathways. In this study, 151 active components, 287 potential targets, and 43 related signaling pathways were predicted. Animal validation tests showed that GDL could effectively improve WD liver lesions, especially by inhibiting the apoptosis of liver tissues.

## Figures and Tables

**Table 1:** The details of genes wih the node degree > 70.

Gene name	Node degree	Gene description
INS	160	Insulin
ALB	152	Albumin
AKT1	152	AKT serine/threonine kinase 1
IL6	150	Interleukin 6
TP53	128	Tumor protein p53
TNF	125	Tumor necrosis factor
EGFR	111	Epidermal growth factor receptor
EGF	100	Epidermal growth factor
ESR1	100	Estrogen receptor 1
STAT3	98	Signal transducer and activator of transcription 3
IL1B	94	Interleukin 1 beta
IGF1	92	Insulin-like growth factor 1
PTGS2	92	Prostaglandin-endoperoxide synthase 2
CCL2	90	C–C motif chemokine ligand 2
IL10	88	Interleukin 10
PPARG	87	Peroxisome proliferator-activated receptor gamma
NOS3	86	Nitric oxide synthase 3
FOS	86	Fos proto-oncogene, AP-1 transcription factor subunit
CAT	82	Catalase
FGF2	82	Fibroblast growth factor 2
LEP	77	Leptin
EDN1	77	Endothelin 1
MAPK14	75	Mitogen-activated protein kinase 14
PTEN	75	Phosphatase and tensin homolog
SIRT1	73	Sirtuin 1
BDNF	72	Brain derived neurotrophic factor
AGT	71	Angiotensinogen
CRP	71	C-reactive protein
AR	71	Androgen receptor

## Data Availability

The datasets generated and/or analyzed during the current study are available from the corresponding author on reasonable request.
